# SCP4ssd: A Serverless Platform for Nucleotide Sequence Synthesis Difficulty Prediction Using an AutoML Model

**DOI:** 10.3390/genes14030605

**Published:** 2023-02-28

**Authors:** Jianqi Zhang, Shuai Ren, Zhenkui Shi, Ruoyu Wang, Haoran Li, Huijuan Tian, Miao Feng, Xiaoping Liao, Hongwu Ma

**Affiliations:** 1 College of Biotechnology, Tianjin University of Science & Technology, Tianjin 300308, China; 2 Biodesign Center, Key Laboratory of Engineering Biology for Low-Carbon Manufacturing, Tianjin Institute of Industrial Biotechnology, Chinese Academy of Sciences, Tianjin 300308, China; 3 National Technology Innovation Center of Synthetic Biology, Tianjin 300308, China; 4 University of Chinese Academy of Sciences, Beijing 100049, China; 5 Tianjin Institute of Industrial Biotechnology, Chinese Academy of Sciences, Tianjin 300308, China; 6 Haihe Laboratory of Synthetic Biology, Tianjin 300308, China

**Keywords:** DNA synthesis, machine learning, AutoML, feature reduction, cloud platform

## Abstract

DNA synthesis is widely used in synthetic biology to construct and assemble sequences ranging from short RBS to ultra-long synthetic genomes. Many sequence features, such as the GC content and repeat sequences, are known to affect the synthesis difficulty and subsequently the synthesis cost. In addition, there are latent sequence features, especially local characteristics of the sequence, which might affect the DNA synthesis process as well. Reliable prediction of the synthesis difficulty for a given sequence is important for reducing the cost, but this remains a challenge. In this study, we propose a new automated machine learning (AutoML) approach to predict the DNA synthesis difficulty, which achieves an F1 score of 0.930 and outperforms the current state-of-the-art model. We found local sequence features that were neglected in previous methods, which might also affect the difficulty of DNA synthesis. Moreover, experimental validation based on ten genes of *Escherichia coli* strain MG1655 shows that our model can achieve an 80% accuracy, which is also better than the state of art. Moreover, we developed the cloud platform SCP4SSD using an entirely cloud-based serverless architecture for the convenience of the end users.

## 1. Introduction

As one of the core technologies of synthetic biology, DNA synthesis, which refers to a group of methods used to construct and assemble de novo nucleotide sequences, has been widely used to construct genetic systems [1,2], artificial synthetic genomes [3], engineered protein sequences [4], and vaccines [5,6]. The first complete gene (a yeast tRNA) was successfully synthesized in 1972, and the first entire chromosome was synthesized in 2014 [7]. JCVI-Syn3A, an updated version of the world’s first artificial organism, was also constructed with the aid of DNA synthesis [8]. In 2021, there were seven COVID-19 mRNA candidate vaccines that were synthesized in vitro from a DNA template encoding either the full-length S protein or the RBD of SARS-CoV-2 [9].

However, DNA sequences cannot always be easily synthesized due to the fact that there are many latent determinants in the sequence that might introduce errors. For example, DNA sequences with a high GC content contain guanine molecules that are stacked together and may affect the annealing process of the DNA molecules [10,11]. In addition, the presence of oligonucleotide secondary structures will also affect the DNA assembly. For example, highly complementary repeats make it almost impossible to assemble multiple fragments accurately, and synthesis usually fails or generates nonspecific products [12,13,14]. In addition to these known factors, some other sequence determinants might also cause DNA synthesis and assembly to fail, and it is still very difficult to evaluate the complexity of the overall sequence synthesis [15,16]. Accordingly, an algorithm that can accurately predict the DNA synthesis difficulty based on broader sequence determinants would be beneficial to reduce the experimental failure rate and help biologists adjust the synthesis strategy if necessary.

A previous study [17] developed a random forest classifier based on nine nucleotide sequence features, named Synthesis Success Calculator (SSC). SSC has a relatively high predictive performance (F1 score of 0.928), but it can be further improved in the following aspects. First, all nine features are related to GC/repeats/hairpins, and other biophysical features are not considered. Secondly, the random forest model could be further improved using other models, such as the ensemble model. Thirdly, it is not straightforward to reproduce the SSC results using the code in their public GitHub repository, and their web application requires user login and contains bugs in multiple sequence predictions (not all nine features are calculated if multiple sequences are submitted, and it will produce wrong predictions based on these features), which makes it inconvenient for end-users.

In this study, we propose a new automated machine learning (AutoML) approach to predict the DNA synthesis difficulty based on 426 initial nucleotide sequence features, which were subsequently reduced to 31 key features with extensive feature-reduction experiments. Interestingly, we found that some sequence features that are not considered by SSC nevertheless have a great impact on the synthesis difficulty. The benchmark results of an unseen test set of 269 sequences show that our methods can achieve a better performance (F1 score of 0.930). Furthermore, experimental validation of ten randomly chosen genes from *E. coli* strain MG1655 also shows that our model outperforms the state-of-the-art model. We provide a downloadable standalone version (https://github.com/tibbdc/scp4ssd) of our model as well as the web application SCP4SSD (https://scp4ssd.biodesign.ac.cn) for sequence synthesis difficulty prediction. The web application has an entirely cloud-based serverless architecture, offering high reliability, robustness, and scalability.

## 2. Materials and Methods

### 2.1. Datasets and Feature Extraction

The raw dataset was collected from the literature [17] and contains 1076 sequences with labels. Unlike the previous study using 38 sequence features (reduced to 9 features in the final model), in this study, 426 sequence features were considered, including 84 composition features [18], 22 accumulated nucleotide frequency features [19], 40 electron-ion interaction potential features [20], 112 K-mer features [21], 15 repeat sequence features, 8 GC content features [22], 3 melting temperature features [23], 7 secondary structure features [24], 20 specific sequence features [25], 114 restriction site features, and the sequence length (Table S1). For each feature, normalization was performed using the sklearn MinMaxScaler function. A more detailed description of the 426 sequence features, which include the 38 features reported in the previous study, is presented in the Supplementary Notes.

SSC adopts a 575–250–251 train–validate–test split. We were having a problem reproducing the same split using the public code. In addition, 251/1076 is not a widely used split. Therefore, in this study, we used the sklearn [26] train_test_split function to divide the data set by 3:1 for training and testing with ‘random_state’ set to 0, which is more commonly used. The 1076 sequences were split into two subsets: 807 sequences were used for training, and 269 sequences were kept unseen for testing.

### 2.2. Evaluation Metrics

F1 scores (F1), Matthew’s correlation coefficient (MCC), and Cohen’s kappa (CK) were used as evaluation metrics to assess the performance of the classifier [26]. The F1 score, a harmonic average of model accuracy and recall, is a widely used measure of binary classification. Matthew’s correlation coefficient considers true/false positives and negatives and is generally regarded as a balanced measure that can be used even if the classes are of very different sizes. Cohen’s kappa is a scoring index that can explain the randomness in the classifier. Their definitions are as follows:

$$F_{1}=\frac{2TP}{2TP+FP+FN}$$

$$MCC=\frac{TP\times TN-FP\times FN\;}{\sqrt{\left(TP+FP\right)\left(TP+FN\right)\left(TN+FP\right)\left(TN+FN\right)\;}}$$

$$CK=\frac{Po-Pe}{1-Pe}$$

$$Po=\frac{TP+TN\;}{TP+TN+FP+FN\;}$$

$$Pe=\frac{\left(TP+FN\right)\times \left(TP+FP\right)+\left(TN+FP\right)\times \left(TN+FN\right)\;}{{\left(TP+TN+FP+FN\right)}^{2}}$$

where TP is the true positive, FP is the false positive, TN is the true negative, FN is the false negative.

### 2.3. Baseline Model Construction

Constructing a baseline model is a common practice in machine learning and data science. The primary purpose of a baseline model is to provide a simple, but reasonable, benchmark for evaluating the performance of more complex models that are developed later. A baseline model is typically a simple and easily interpretable model that is developed using a general method or algorithm, such as linear regression or logistic regression. The model is developed without any feature engineering, hyperparameter tuning, or other optimization techniques. The performance of this model can then be used as a reference point for comparing the performance of other, more complex models that are developed later.

AutoML, or automated machine learning, is a set of techniques that automate many of the tedious and time-consuming aspects of building machine learning models, such as hyperparameter tuning, feature selection, and model selection. AutoML has recently achieved substantial success [27,28]. Auto-sklearn is a popular open-source AutoML tool that is based on the machine learning library scikit-learn [29].

In this study, we constructed a baseline ensemble model with all 426 features using Auto-sklearn, which automatically selected the best combination of models and hyperparameters to use in the ensemble based on the provided data and the given machine learning task. The model integrates 16 classical machine learning methods, including random forest, AdaBoost, Bernoulli naive Bayes, decision tree, k-nearest neighbors, and linear support vector machine.

### 2.4. Feature Reduction

Feature reduction is an important technique in machine learning, as it can improve the performance of models by removing noisy or irrelevant features and reducing the redundancy. Noisy or irrelevant features are features that do not have a significant impact on the outcome of the model but may still be included in the dataset. These features can cause the model to overfit the training data and perform poorly on new, unseen data [30]. By removing these redundant features, the model can be simplified and made more interpretable, which can aid in understanding the underlying relationships between the input variables and the output.

In this study, we chose three popular feature-selection algorithms for feature reduction: genetic algorithm, correlation coefficient selection method, and variance selection method [30,31]. For each criterion, we selected different cutoffs after checking the distributions. For the correlation, we chose 0.1, 0.15, 0.2, and 0.3, while for the variance, 0.01, 0.02, 0.03, and 0.04 were chosen. For the genetic algorithm, we chose 10,000 or 100,000 rounds.

To obtain the best model with a reduced number of features, we extensively tested the combinations of cutoff settings. More specifically, we designed 74 computational experiments, including 10 experiments considering only one method, 32 experiments considering the combination of two methods, and an additional 32 experiments considering the combination of all three methods (Table S2). We performed 10 independent repeats for each experiment to calculate the average metrics.

### 2.5. Model Training and Calculation of Feature Importance

After feature reduction, we trained our final classifier. Finally, we wrapped all features, parameters, and statistical model components to build a production model for final deployment. We used the inspection module of scikit-learn [26] to calculate the feature importance, which defines the decrease in a model score when a given feature is randomly permuted. Thus, a higher score indicates a higher dependence of the model’s predictions on the tested feature. We also calculated the partial dependence for each feature [26].

### 2.6. Experimental Validation

Ten genes from the genome of *E. coli* MG1655 were chosen for experimental validation. To verify the reliability of the methods, we considered two requirements when selecting genes. One is the case where the predictions of two methods are consistent, and the other is the case where the predictions of two methods are inconsistent. For the case where the prediction results are consistent, we randomly selected 3 genes. In addition, for the case where the prediction results are inconsistent, we make a random selection on the premise of balancing the proportion as much as possible. Specifically, we randomly picked 7 genes for which our prediction differed from the SSC prediction.

In total, we randomly picked five genes that were predicted (SSC) to be difficult to synthesize and five genes that were predicted (SSC) to be easy to synthesize. We mixed the oligonucleotides in equimolar concentrations and first preassembled the 10 genes, respectively, using a polymerase cycling assembly (PCA) reaction, followed by amplification of the DNA fragments using polymerase chain reaction (PCR). The reaction products were analyzed with gel electrophoresis to check the purity of the target fragment and possible dimers.

## 3. Results and Discussions

### 3.1. Baseline Model Construction

First, we constructed a baseline ensemble model with all 426 features using Auto-sklearn. A crucial feature that affects the performance of Auto-sklearn is the resources (memory and time) that the scikit-learn algorithm is allowed to use. In this study, we set it to 10 min according to a previous study [29]. In addition, the Auto-sklearn approach was based on the Bayesian framework, and the final prediction model was slightly different each time. Therefore, we conducted 10 iterations to account for randomness. The lowest F1 score of the ten runs was 0.917, and the highest was 0.936. The final mean of the F1 score was 0.925, and the standard deviation was 0.0057.

### 3.2. Feature Reduction

To remove irrelevant and redundant features, we chose three widely used feature-selection algorithms for feature reduction based on correlation coefficient selection, variance selection, and a genetic algorithm. As shown in the materials and methods section, we set different selection criteria for each method and designed 74 computational experiments to find the best feature combinations, including ten experiments considering only one method, 32 experiments considering the combination of two methods, and an additional 32 experiments considering the combination of all methods.

**Figure 1:**
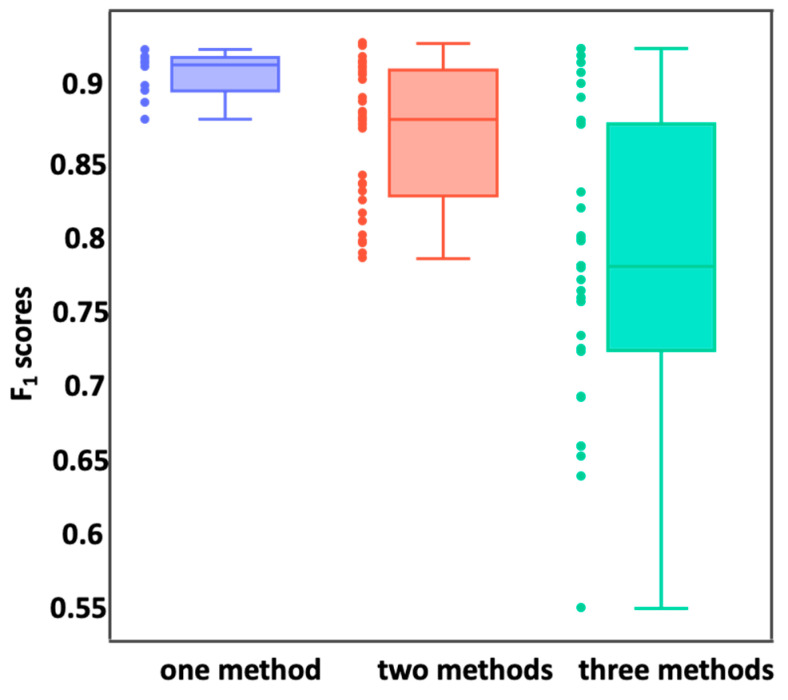
Boxplot of F1 scores. The blue dots represent the F1 scores of the models containing the reduced features obtained using a single method. The red dots represent the F1 scores of the models containing the reduced features obtained using the combination of two methods. The green dots represent the F1 scores of the model containing the reduced features obtained using the combination of three methods.

As shown in Figure 1, with the combination of more feature-selection methods, fewer features were included, and the final performance was likely to decline. When only one feature-reduction method was used, the highest F1 score was 0.922 with the variance cutoff set to 0.01 (Figure S1). This reduced model contained 304 features, which was still too many. The same correlation between the performance and the cutoffs was observed as well (Figure S1). Accordingly, the models with fewer features will likely have a poorer performance.

Interestingly, we found that some models with very few features can achieve a very good performance. When a combination of two feature-selection methods was used, we found that the ensemble model based on the combination of the correlation coefficient 0.2 and 100,000 rounds of the genetic algorithm (31 features) performed the best, with an F1 score as high as 0.930 (Figure S2). In the case of the combination of all three methods, the highest F1 score was 0.923, and the corresponding model had 30 features (Figure S3). Therefore, we chose the combination of 31 features since it showed the best performance. In this combination of 31 features, there are 25 features uniquely found in our work (Figure S4 and Table S3) and six features among the 38 features of the previous work.

### 3.3. Benchmark Results of SCP4SSD and SSC

The final ensemble model was composed of 16 additional trees, five random forests, one AdaBoost, one latent Dirichlet allocation, and one support vector machine with a linear kernel (Table S4 describes the weights of each component). As shown above, random forest models, adopted in the SSC, are already included in our model, and the additional models will probably improve the average robustness or reliability of the ensemble model.

**Figure 2:**
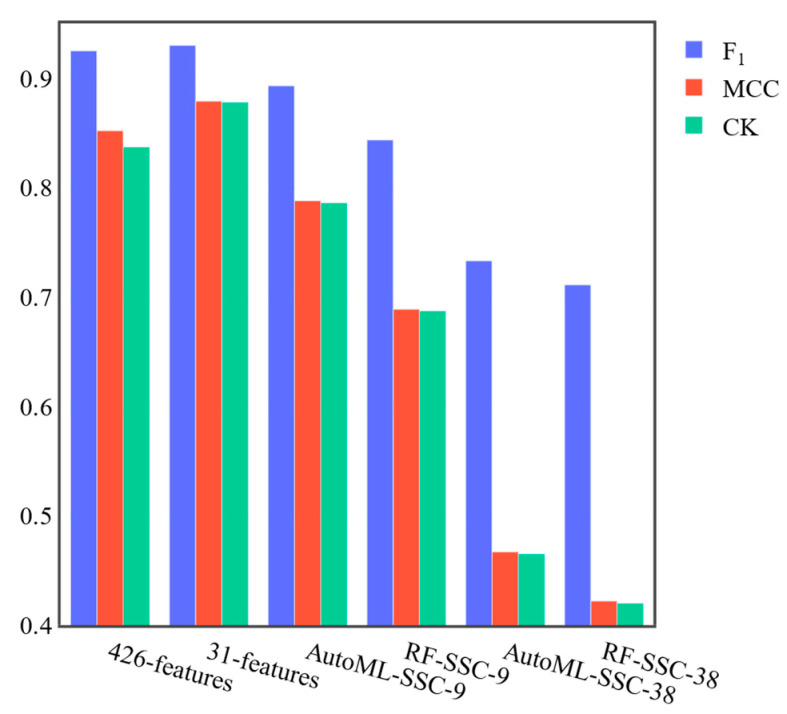
Comparison of our model with SSC. ‘426-features’ is our baseline model with 426 features; ‘31-features’ is our final model; ‘AutoML-SSC9’ is the ensemble model based on 9 features from SSC; ‘RF-SSC-9’ is the random forest model based on 9 features from SSC; ‘AutoML-SSC38’ is the ensemble model based on 38 features from SSC; ‘RF-SSC-38’ is the random forest model based on 38 features from SSC.

The F1 score of SSC in the previous study was 0.928. As mentioned in the materials and methods section, we had a problem reproducing the same 575–250–251 train–validate–test split with the public code and could not reproduce the results. In addition, SSC has a model hyperparameter optimization process in which all 1076 sequences are used for training. This process would boost the overall performance and the SSC might be overfitted. In order to make a fair comparison, we built four models, including a random forest model using the 9/38 features reported in the SSC and an ensemble model using 9/38 features reported in the SSC. All of them use the same dataset split as described in this study. For the case of the nine features, the F1 score was 0.843, which was lower than the 0.928 reported in the literature. With the ensemble model using AutoML, the F1 score was 0.893 (Figure 2). This indicates that the ensemble model is indeed better than the random forest model solely for predicting the sequence synthesis difficulty.

As shown in Figure 2, our model outperformed the SSC or ensemble model with SSC features in terms of all metrics. More specifically, our method had the highest F1 score of 0.930 (std 0.007), MCC score of 0.879 (std 0.010), and CK score of 0.878 (std 0.004). In contrast, the random forest model with the nine sequence features had an F1 score of 0.843, an MCC score of 0.689, and a CK score of 0.687. The ensemble model based on the nine features reported in the SSC had an F1 score of 0.893 (std 0.007), an MCC score of 0.788 (std 0.009), and a CK score of 0.786 (std 0.006). The random forest model with the 38 sequence features had an F1 score of 0.711, an MCC score of 0.422, and a CK score of 0.420. Finally, the ensemble model based on 38 features had an F1 score of 0.733 (std 0.0114), an MCC score of 0.467 (std 0.003), and a CK score of 0.465 (std 0.005). Interestingly, consistent with the previous work, we also found that the model with nine features is better than the model with 38 features using both random forest and ensemble models.

**Figure 3:**
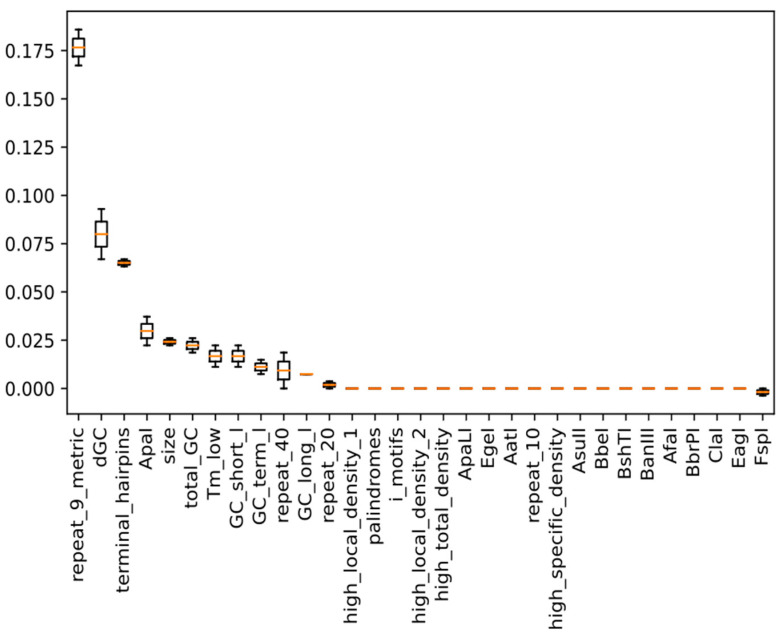
The relative importance of all 31 features.

We also conducted further feature-importance calculations. Interestingly, as shown in Figure 3, among the top 12 significant features with positive contributions, we found some features that were not considered by SSC (Figure 3). For example, ‘dGC’, representing the fluctuation of GC content within a 100 bp sliding window, was the second-most significant feature. A large fluctuation of the GC content within regions may affect the chain extension in the process of oligonucleotide synthesis. The restriction site of ApaI (‘GGGCCC’) is the fourth-most significant feature. This restriction site has an extremely high GC content, and if the same pattern repeats many times, it might affect DNA synthesis (Figure S5). Moreover, the number of 20 bp sliding windows with low GC content (‘GC_short_l’) might also affect the synthesis of the nucleotide sequence. In our model, three important local GC content features (‘dGC’ ranked 2th, ‘GC_short_l’ ranked 8th, ‘GC_long_l’ ranked 11th, Figure 3) were all low-GC-content features. This might imply that local fragments with low GC content might have a more important impact than fragments with high GC content.

### 3.4. Experimental Validation

The sequence prediction results of 4318 sequences in the *E. coli* strain MG1655 genome showed that our model predicted 3777 easy-to-synthesize sequences, accounting for 87%, and 541 difficult-to-synthesize sequences, accounting for 13%. SSC predicted that 15.1% of the sequences in *E. coli* were difficult to synthesize. Both our and SSC models find that most genes are easy to synthesize.

**Figure 4:**
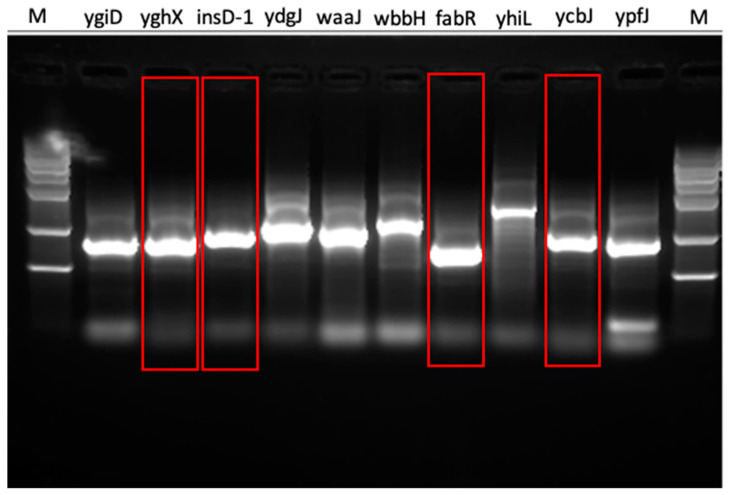
Experimental validation of 10 genes from *E. coli*. Four genes that can be easily synthesized are marked in red boxes.

To further verify the reliability of our model, especially for those difficult-to-synthesize genes, we randomly picked five genes that were predicted to be difficult to synthesize and five genes that were predicted to be easy to synthesize by SSC (details in Section 2.6). The experimental results show that out of the ten selected sequences (Table S5), the synthetic sequences of the *yghX*, *insD-1*, *fabR*, and *ycbJ* genes had very few impurity bands and possible dimers (Figure 4), indicating that these four sequences are indeed easy to synthesize. Overall, our method gave eight correct predictions for these ten genes, while SSC only made five accurate predictions (Table 1).

**Figure 5:**
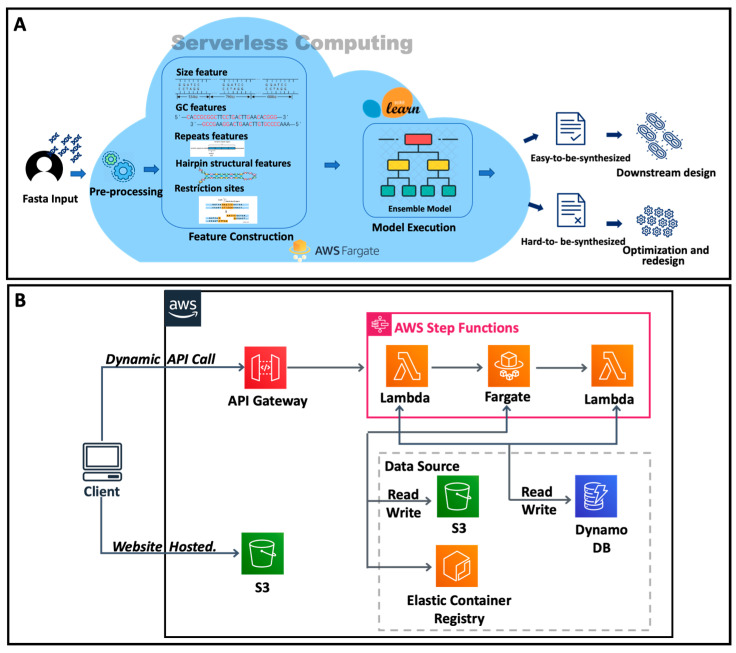
(**A**) The workflow of SCP4SSD; (**B**) The architecture of SCP4SSD.

Our method predicted that the *waaJ* and *yhiL* gene sequences are difficult to synthesize, while SSC predicted the opposite. After checking the features, we found that the three important features ‘Tm_low’, ‘GC_short_l’, and ‘GC_long_l’ have high values in these two genes. Both ‘Tm_low’ and ‘GC_short_l’ are considered by SSC as well. However, ‘GC_long_l’ is missing from SSC and these two genes have 268 or 183 100 bp sliding windows with less than 30% GC content, respectively. Moreover, the overall scores of DNAWorks for these two genes were above 40, with repeat/mispriming/GC scores indicating that these two genes are difficult to synthesize [32]. The gel electrophoresis also showed many impurity bands for these two genes (Figure 5). This finding implies that our method captures some important features missed by SSC.

Conversely, our method predicted that the two genes *yghX* and *insD-1* are easy to synthesize, while SSC predicted the opposite. For these two genes, the gel electrophoresis shows clear target bands and no dimers (Figure 4). In addition, the DNAWorks scores for both these genes were relatively low, which was consistent with our predictions.

### 3.5. Cloud Platform

To facilitate easy access for end-users, we developed the cloud platform SCP4SSD (Figure 5A), which was built using the serverless architecture that has emerged in recent years [33]. The main advantage of this architecture is that when users submit tasks through the front-end, each submitted task will create a virtual server to respond to the request, and it will be automatically released after the analysis is finished [34]. This technology helps us control and save costs, paying only for what we use [35]. On the other hand, when the concurrence increases, it can compute in parallel and guarantee a stable service level.

The website was developed using React and is hosted on AWS S3 (Figure 5B). When end-users submit sequences in FASTA format, the website first checks the sequences and then upload them to the S3 bucket. The file path is passed to the AWS step functions via the API gateway. We used AWS step functions to manage the AWS Fargate and AWS Lambda for the backend (Figure 5B). AWS Fargate is used to preprocess the input of the sequences, predict the synthesis difficulty for the query sequences, and upload the result files to AWS S3. After that, another AWS Lambda function is invoked to check the status of AWS Fargate and update the DynamoDB database for tracking the job ID and status. This process is conducted in parallel for each submission, regardless of how much demand there is on the website, showcasing the usefulness of serverless computing. We use browser cookies to record the job ID so that users can view the previously submitted records (within 7 days) without login. Our platform will report the prediction results (whether it is easy to be synthesized: yes or no). In addition, the platform will report the relevant sequence determinants that were considered by the model so that users can intuitively understand the sequence features that affect DNA synthesis.

## Figures and Tables

**Table 1:** Comparison of our methods with SSC based on 10 genes in *E. coli*.

Gene Symbol	DNAWorks Score	Our Method	SSC	Experimental Result
*ygiD*	6.485	EASY	HARD	HARD
*yghX*	6.756	EASY	HARD	EASY
*insD-1*	4.512	EASY	HARD	EASY
*ydgJ*	6.5	HARD	EASY	HARD
*waaJ*	54.875	HARD	EASY	HARD
*wbbH*	64.955	HARD	HARD	HARD
*fabR*	6.084	EASY	EASY	EASY
*yhiL*	41.894	HARD	EASY	HARD
*ycbJ*	9.622	EASY	EASY	EASY
*ypfJ*	6.625	EASY	HARD	HARD

## Data Availability

The code and test dataset are available on GitHub https://github.com/tibbdc/scp4ssd.

## Supplementary Materials

The following supporting information can be downloaded at: https://www.mdpi.com/article/10.3390/genes14030605/s1. Figure S1: Box distribution of F1 scores for feature reduction by single method. (a) F1 score distribution of single variance selection method. (b) F1 score distribution of single correlation coefficient method. (c) F1 score distribution of single genetic algorithm. Figure S2: Box distribution of F1 scores of feature reduction by two combination methods. (a) F1 score distribution of correlation coefficient method and genetic algorithm. (b) F1 score distribution of variance selection method and genetic algorithm. (c) F1 score distribution of correlation coefficient method and variance selection method. Figure S3: Box distribution of F1 scores of feature reduction by all methods. (a) The F1 score distribution of 10,000 rounds of genetic algorithm and the other two methods. (b) The F1 score distribution of 100,000 rounds of genetic algorithm and the other two methods. Figure S4: The Venn diagram shows the relationship among different sets of features. Figure S5: Partial dependence for top 4 features. Table S1: 426 Feature descriptions. Table S2: Experiments of feature selection. Table S3: Comparison of SSC and our method. Table S4: Components of the final ensemble model. Table S5: 10 selected genes for experimental verification. Supplementary Notes, S1: 84 composition features; S2: 22 accumulated nucleotide frequency features; S3: 40 electron-ion interaction potential features; S4: 112 k-mer features; S5: 15 repeat sequence features; S6: 8 GC content features; S7: 3 melting temperature features; S8: 7 secondary structure features; S9: 20 specific sequence features; S10: 114 restriction site features; S11: Sequence length.
