# Supplementary material for: SCP4ssd: A Serverless Platform for Nucleotide Sequence Synthesis Difficulty Prediction Using an AutoML Model
Source: Genes (Basel). 2023 Feb 28;14(3):605. doi: 10.3390/genes14030605 (PMC10048150; doi:10.3390/genes14030605)
Supplement: Supplementary file 1 [file genes-14-00605-s001.zip › Supplementary Notes.docx]

**Supplementary Notes**

Many factors have a crucial impact on the difficulty of DNA sequence synthesis, especially some biological features of the sequence. In this work, we focus on collecting some relatively relevant biological features, belonging to a total of 11 categories, and subdivide them in each category by different coding methods, such as the setting of different sliding windows, the selection of different thresholds, and so on. Finally, we collected 426 sequence features as feature input, including 84 component features, 22 amino acid frequency features, 40 electron-ion interaction potential features, 112 k-mer features, 15 repetitive segment features, 8 GC content features, 3 Tm value-related features, 7 secondary structure features, 20 sequence-specific structural rule features, 114 restriction site features, and 1 sequence length feature, These features are all implemented by Python code, and 426 features are jointly digitized by using the call of computing software such as VinneaRNA and Biopython. The detailed meaning and construction method of each type of feature are as follows:

S1. 84 composition features

The influence of local nucleotide content on the difficulty of DNA synthesis is considered in the composition features. We set different sliding windows, namely the average, minimum, and maximum contents of A, T, G, and C nucleotides under the 5bp, 10bp, 15bp, 25bp, 30bp, 50bp and 75bp windows, which totally contain 84 features.

S2. 22 accumulated nucleotide frequency features

Accumulated nucleotide frequency features consider the effect of overall and local nucleotide frequency on the difficulty of DNA synthesis. Among them, the cumulative nucleotide frequency and average cumulative nucleotide frequency calculated for the whole sequence can represent the overall nucleotide frequency features; In addition, through different sliding windows, namely 5bp, 10bp, 15bp, 20bp, 25bp, 30bp, 40bp, 50bp, 60bp and 70bp, the cumulative nucleotide frequency and the average nucleotide frequency are calculated respectively, including 22, which can represent the local nucleotide frequency features.

S3. 40 electron-ion interaction potential features

The features of electron-ion interaction potential take into account the influence of the interaction between bases on the difficulty of DNA synthesis. According to the literature, the action potential value of nucleotide A is 0.1260; The action potential value of nucleotide T is 0.1335; The action potential value of nucleotide C is 0.1340; The action potential value of nucleotide G is 0.0806. We divide the electron-ion interaction potential into four different situations according to different thresholds: super strong (value higher than 0.131), extremely strong (value higher than 0.130), sub-strong (value higher than 0.129), and slightly strong (value higher than 0.127). Through different sliding windows, namely 3bp, 5bp, 10bp, 15bp, 20bp, 25bp, 35bp, 50bp, 60bp, and 70bp, we count the above four situations respectively. In total, there are 40 features representing the interaction potential.

S4. 112 k-mer features

The k-mer feature considers whether there is a specific content of motif, that is, a special sequence, which affects the difficulty of DNA synthesis. We have selected several different motif combinations of 2-mer, 3-mer, 4-mer, 5-mer, 6-mer, 7-mer, 8-mer, 9-mer. Taking 2-mer as an example, because there are four amino acid types, the meaning it represents is the combination of two linked amino acids, including 16 motifs in total, and the other motif combinations increase exponentially. Because the sequence length is different between sequences, we do not count the number of motifs, but calculate the proportion of each motif in the whole sequence, and generate features. Features include the total proportion of each phantom under N-mer, the average proportion of each phantom, the proportion of the largest phantom, the proportion of the smallest phantom, and the digital scale features 1-9 times higher than the average proportion of the phantom (the number of the higher than n times multiplied by 2n), including a total of 112 features.

S5. 15 repeat sequence features

The feature of the repeat segment takes into account the influence of the repeat in the sequence on the difficulty of DNA synthesis. First, we use Python language to calculate the repetitive fragments contained in a nucleotide sequence and then generate features. These features include 15 types, which are the number of repetitive segments with the highest frequency, 9-mer (the number of segments with repeats greater than or equal to 9 bp * 9/sequence length), the number of windows composed of repetitive segments with more than 90% of nucleotides under the 70 bp window, the number of windows composed of repetitive segments with more than 60% of nucleotides under the 500 bp window, and the number of specific repeats with 40% of the total sequence length, Whether 69% of the nucleotides of the whole sequence participate in any repeat (yes, mark 1, no mark 0), the length of the maximum repeat segment, the number of repeat segments less than or equal to 10 bp, the number of repeat segments greater than 10 bp less than or equal to 15 bp, the number of repeat segments greater than 15 bp less than or equal to 20 bp, the number of repeat segments greater than 20 bp less than or equal to 25 bp, the number of repeat segments greater than 25 bp less than or equal to 40 bp, and the number of repeat segments greater than 40 bp, The number of repeats within 60 bp at both ends of the sequence is more than 10 bp, which is more than 5 bp and the adjacent interval is not more than 5 nucleotides.

S6. 8 GC content features

The GC content features consider the influence of the overall and local GC abundance in the sequence on the difficulty of DNA synthesis. The proportion of GC content to the number of nucleotides in the whole sequence is taken as the overall feature, and the GC content in different sliding windows is set as the local feature. They include: the number of 100 bp windows with GC content higher than 70% and the number of 20 bp windows with GC content higher than 80% to measure the high GC abundance in the sequence; The number of 100bp windows with GC content less than 30% and the number of 20bp windows with GC content less than 20% are used to measure the lower GC abundance in the sequence; The number of 20 bp windows with GC content higher than 70% and less than 30% in the range of 50 bp at both ends of the sequence is used to measure the GC abundance at both ends of the sequence; Calculate the number of 100bp windows in which the GC content of 20bp sub-window changes more than or equal to 50% within the range of 100bp window to measure the local GC change range, including 8 features.

S7. 3 melting temperature features

The melting temperature feature take into account the influence of the unwinding temperature of the sequence neutron sequence on the difficulty of DNA synthesis. It is characterized by three features, including: calculating the number of 20bp subsequence windows with Tm value greater than or equal to 70, calculating the number of 20bp subsequence windows with Tm value less than or equal to 40, and calculating the number of 100bp windows with Tm value change greater than or equal to 30 in any 20bp subsequence window.

S8. 7 secondary structure features

The secondary structure feature considers the possible hairpin structure in the sequence and the influence of palindrome sequence on the difficulty of DNA synthesis. First, the secondary structure that may exist in the sequence is calculated by calling VinneaRNA2.1 and characterized by different features, including 7 features. They include the number of hairpin structures whose stem length is greater than or equal to 20 bp in the sequence; The length of the longest stem in the hairpin structure in the sequence; The number of palindromes in the sequence; The highest GC content of all hairpin structures in the sequence; The GC content in the sequence is 80% of the number of hairpin structures; The number of hairpin structures in the 60bp window at both ends of the sequence; The number of hairpin structures with 17 consecutive base pairs in the 100-bp window in the sequence.

S9. 20 specific sequence features

Specific structural rule features consider the effect of special base arrangement in the sequence on the difficulty of DNA synthesis. These include: the number of polynucleotides, the number of polynucleotides and polynucleotides, the number of G-quadruplex structures that may be formed by serially repeated polyguanine, the number of i-motifs that may be formed by serially repeated polycytosine, the number of specific base combinations of i-motifs with biological functions recorded in 15 documents, and the number of CpG islands, a total of 20 features.

S10. 114 restriction site features

The features of restriction endonuclease sites consider the influence of different restriction endonuclease sites on the difficulty of DNA synthesis. We collected a total of 113 different enzymes, calculated the number of restriction site sequences of different enzymes, and the sum of the number of all restriction sites in the summary sequence, resulting in a total of 114 features.

S11. Sequence length

The sequence length feature represents the base number of the nucleotide sequence contained in the sequence, where the bp number of the sequence is calculated.
