# Supplementary figures and images for: SCP4ssd: A Serverless Platform for Nucleotide Sequence Synthesis Difficulty Prediction Using an AutoML Model
Source: Genes (Basel). 2023 Feb 28;14(3):605. doi: 10.3390/genes14030605 (PMC10048150; doi:10.3390/genes14030605)

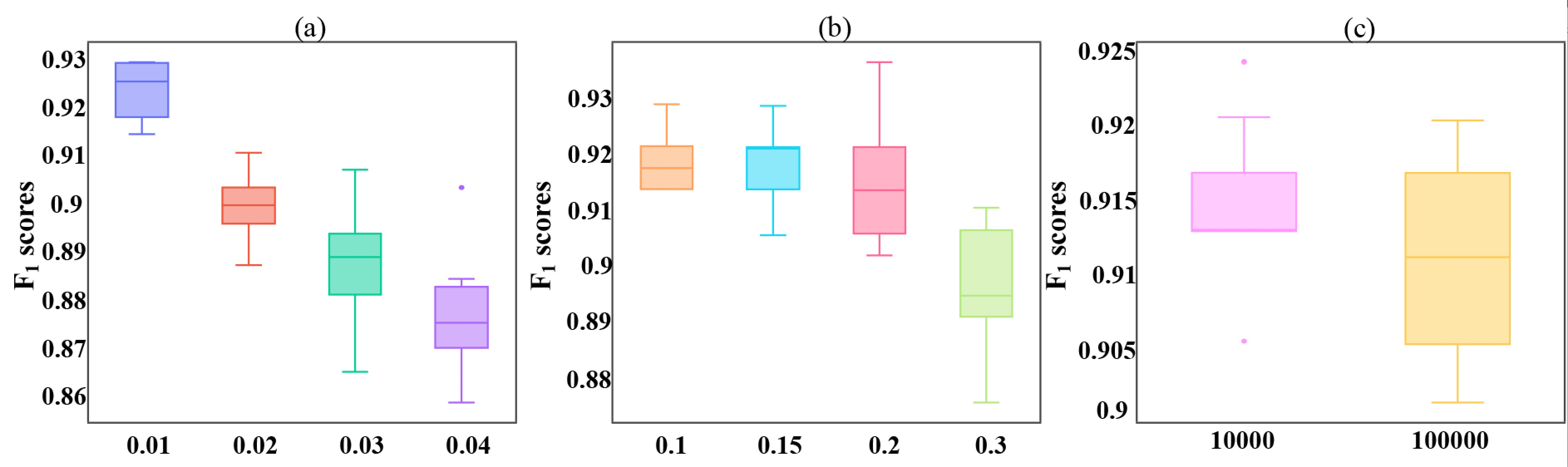

Supplement: Supplementary file 1 [file genes-14-00605-s001.zip › FigureS1.png]

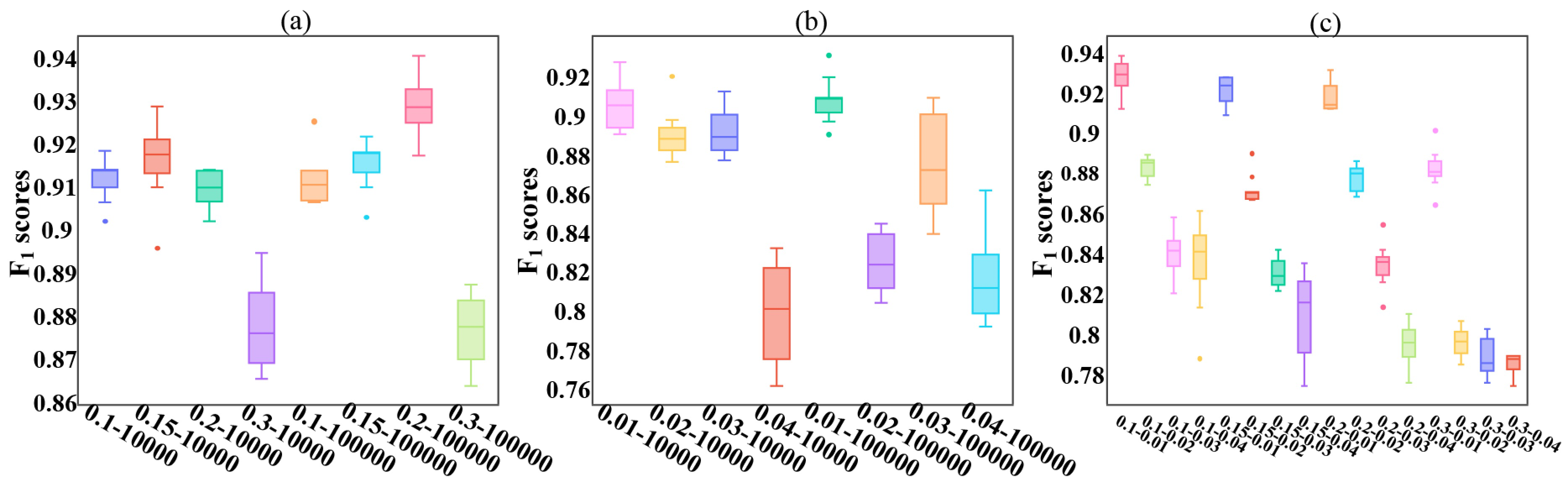

Supplement: Supplementary file 1 [file genes-14-00605-s001.zip › FigureS2.png]

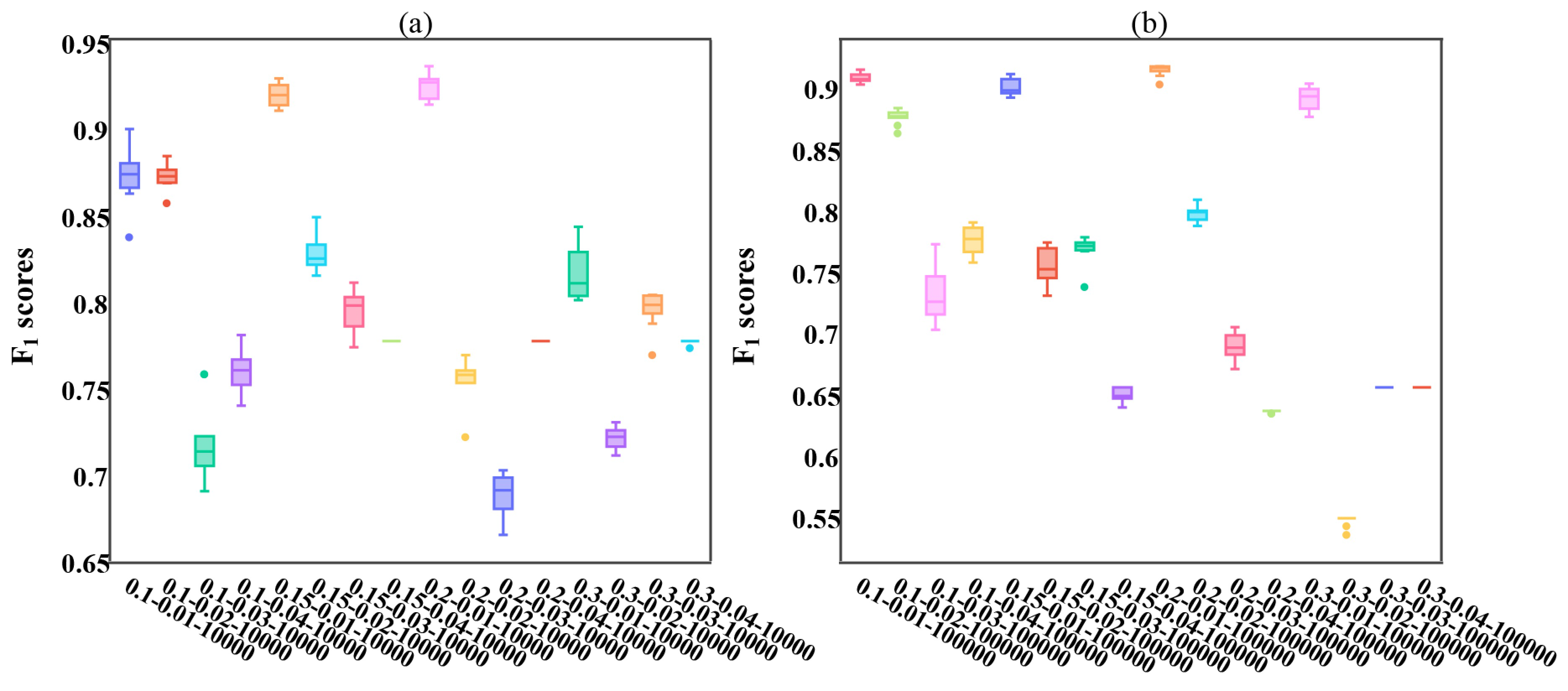

Supplement: Supplementary file 1 [file genes-14-00605-s001.zip › FigureS3.png]

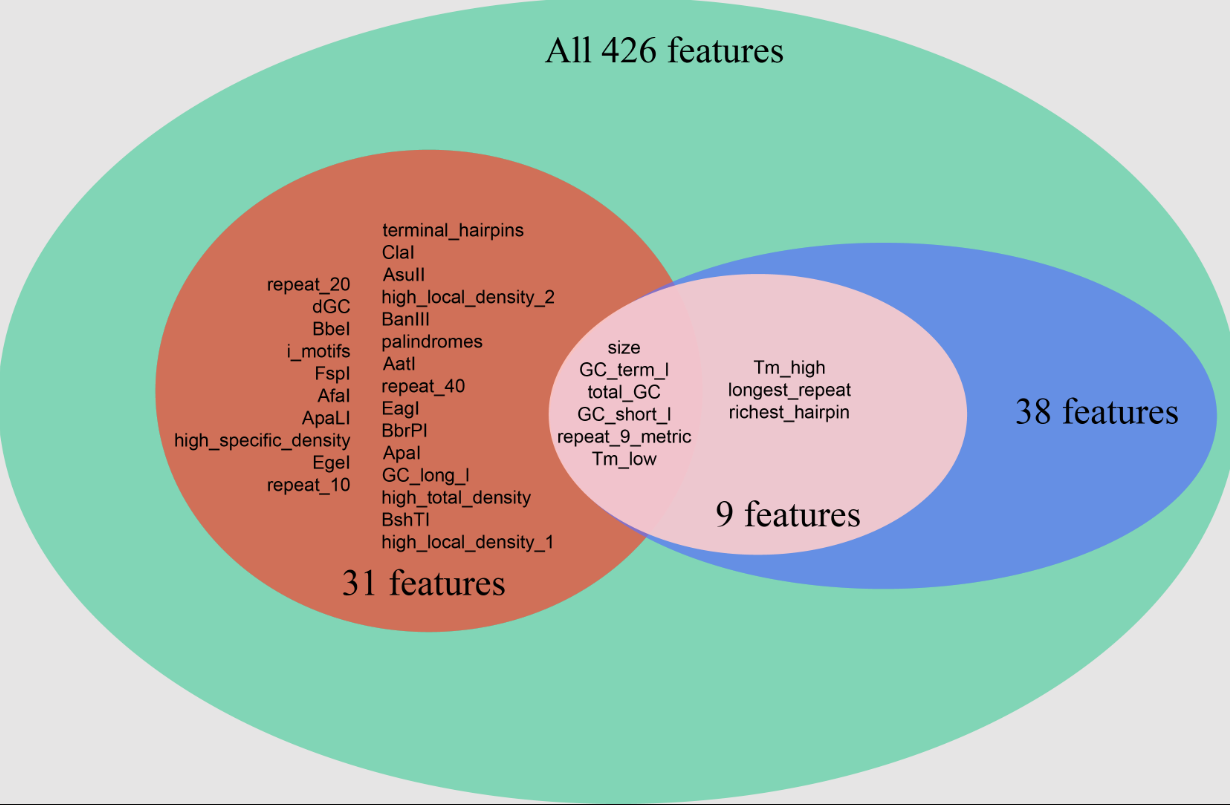

Supplement: Supplementary file 1 [file genes-14-00605-s001.zip › FigureS4.png]

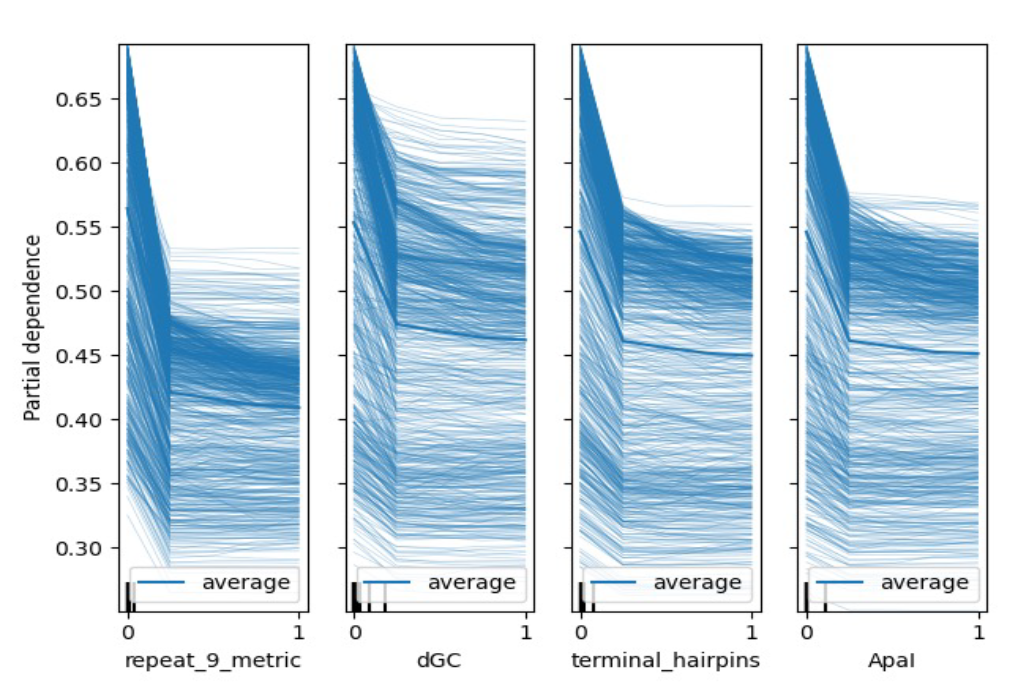

Supplement: Supplementary file 1 [file genes-14-00605-s001.zip › FigureS5.png]
